# The analysis of anxiety and mood in healthy late-reproductive-stage women with regard to hormonal and genetic factors

**DOI:** 10.1007/s00737-016-0667-8

**Published:** 2016-09-10

**Authors:** Anna Jurczak, Małgorzata Szkup, Anna Grzywacz, Krzysztof Safranow, Elżbieta Grochans

**Affiliations:** 1 Department of Nursing, Pomeranian Medical University in Szczecin, ul. Żołnierska 48, 71-210 Szczecin, Poland; 2 Department of Psychiatry, Pomeranian Medical University in Szczecin, Broniewskiego 26, 71-460 Szczecin, Poland; 3 Department of Biochemistry, Pomeranian Medical University in Szczecin, Powstańców Wielkopolskich 72, 70-111 Szczecin, Poland

**Keywords:** Polymorphism, Women, Mood, Anxiety, Reproductive

## Abstract

The purpose of this study was to determine whether anxiety and mood disorders in late-reproductive-stage women are related to the serotonin transporter and monoamine oxidase A gene polymorphisms. Research instrument used in this study were the State-Trait Anxiety Inventory and the UWIST Mood Adjective Checklist. The 44-bp VNTR polymorphism in the 5-HTT (*SLC 6A4*) promoter region and the 30-bp VNTR polymorphism in the *MAO-A* promoter region were analyzed. The study included 345 healthy Polish women in the late reproductive stage. The mean age of the participants was 42.3 ± 4.5 years. State anxiety was observed in 16.8 % of the women and trait anxiety in 14.5 %. There were no statistically significant differences in the mood and the mean levels of anxiety depending on the presence of the polymorphisms analyzed in this study. Depressed mood is frequent among healthy women in the late reproductive stage. Anxiety is definitely less common. The study did not demonstrate the relationship between the 5-HTT and *MAO-A* gene polymorphisms, and the severity of anxiety and mood disorders in healthy late-reproductive-stage women.

## Introduction

The 2001 Stages of Reproductive Aging Workshop (STRAW) standardized the classification of menopause status. A decade later, the development of the knowledge concerning changes in a woman’s body resulted in a new, updated STRAW + 10 version.

The study presented here included women who, according to this classification, were in the late reproductive stage (the −3 stage), i.e., the stage typified by the onset of a decline in fecundability and first changes in the menstrual cycle. In accordance with the STRAW + 10 criteria, this phase is divided into two subphases: −3b and −3a. The length of the menstrual cycle in the −3b subphase remains unchanged. What can be observed, however, is that the levels of anti-Müllerian hormone (AMH) and antral follicle count decrease. The −3a subphase is characterized by a shortening of the menstrual cycle. Moreover, in the early follicular phase, the level of follicle-stimulating hormone (FSH) increases, while the levels of other biomarkers of ovarian aging decrease (Harlow et al. 2012). The level of FSH >30 ml U/ml is regarded as critical and marked by the lack of ovarian follicles in the ovaries. In this period, the level of estradiol decreases dramatically (40–400 pg/ml depending on the phase of the menstrual cycle before menopause to 20–30 pg/ml after menopause) (Baranowski 2004).

Even though they can be related to the menopausal period, symptoms such as depressed mood, nervous tension, and concentration problems are not treated as its typical signs (Banger 2002). Still, as opinion surveys show, a vast majority of women expects the occurrence of these symptoms and is afraid of them (Podgórska 2003). Mood disorders more often affect women than men. As some authors (Krogulski 2004) claim, the perimenopausal period entails escalation of symptoms due to the coexistence of biological and nonbiological factors. Many women feel worried thinking about approaching menopause. They are afraid of physical complaints, but, above all, they treat the climacteric as the end of a full-value life and the beginning of old age (Sprawka et al. 2008).

Women are at a two to three times higher risk of developing anxiety disorders, especially in the perimenopausal period, than men. The study conducted by Weiss et al. (2015) showed that a woman’s level of anxiety was the strongest predictor of her likelihood of having severe depression. The etiology of panic disorder includes the serotonergic, noradrenergic, gamma-aminobutyric acid (GABA), and neuroanatomic models, as well as the genetic hypothesis (the presence of strong stress factors is necessary). In women, environmental and genetic factors can increase anxiety and mood changes. Genes involved in serotonergic pathways are regarded as candidate genes due to the documented role of serotonin (5-HT) in the etiopathogenesis of mood disorders. One of the most frequently analyzed genes of this group is *SLC6A4*—a single gene located on the 17q12 chromosome, encoding the serotonin transporter (5-HTT). The polymorphism of this gene is characterized by the insertion or deletion of a 44-bp fragment and entails different gene transcriptional activity. A short allele (allele with deletion of a 44-bp sequence) is characterized by a three times lower transcriptional activity than a long allele (allele with insertion of a 44-bp sequence) (Hauser and Dmitrzak-Węglarz 2010). Available results show that the presence of the *s* allele of this gene is associated with a higher neuroticism and the development of anxiety and mood disorders (Jurczak et al. 2015; Stein et al. 2009). What is more, when combined with adverse environmental factors, it may increase the probability of mood disorders related to stressful life events (Caspi and Moffitt 2006; Wilhelm et al. 2006; Jacobs et al. 2006; Paaver et al. 2008). Carriers of the *s* allele of this gene are more susceptible of affective disorders and anxiety states (Ebstein 2006; Stein et al. 2008). The monoamine oxidase A (*MAO-A*) gene that encodes the MAO-A enzyme may be also responsible for an inclination to depression. Sabol et al. (1998) were the first to describe the *uMAO-A* polymorphism, which is a variable-number tandem repeat (VNTR) polymorphism in the *uMAO-A* promoter region. It consists of a 30-bp repeated sequence that may be present in 3, 3.5, 4, or 5 copies (Black et al. 1991). The “3” allele is associated with a lower transcriptional activity of the gene, while the “3.5,” “4,” and “5” alleles are related to a higher *MAO-A* activity (Denney et al. 1999). Interesting conclusions were drawn from the research which demonstrated a U-shaped relationship between behavior and the *MAO* activity in thrombocytes. It was found that both individuals with a low and those with a high *MAO* activity show higher impulsivity in neuropsychological tests (Klinteberg et al. 1987). They are significantly more likely to develop nicotine addiction (Harro et al. 2004) and anxiety disorders (Schalling et al. 1987).

Ovarian hormones (FSH, AMH, E2) play a key role in sustaining proper brain function and the brain’s response to environmental stimuli (Toffoletto et al. 2014). It is a common belief that ovarian hormones may be essential for the emergence of differences between individuals of both sexes in the incidence of mood and anxiety disorders. Nevertheless, mechanisms underlying this phenomenon have not yet been fully explicated. It is assumed that ovarian hormones may contribute to the regulation of mood through the mediating effect of psychological stress, which often precedes episodes of depression. It may result in mood disorders, which are especially common among women (Albert et al. 2015).

The aim of this study was to investigate how mood and anxiety of late-reproductive-stage women are related to the 5-HTT and *MAO-A* gene polymorphisms.

## Materials and methods

The study involved 345 healthy, late-reproductive-stage women from northwest Poland. The criteria for inclusion in the study were as follows: no endocrine disorders, no neoplastic diseases, and no psychiatric problems. The criteria for exclusion from the study were abnormal smear test results, diagnosis of thyroid diseases and/or diabetes, diagnosis of neoplastic diseases, and diagnosis of mental diseases.

Women with Axis I mental disorders according to the ICD-10 classification were excluded from the study using the PRIME-MD questionnaire and a psychiatric examination (Spitzer et al. 1999). In all women, the levels of AMH and FSH were determined. On the basis of the results, the women were categorized as being in the late reproductive stage.

All participants gave their informed consent to participate in the research. The study was conducted in accordance with the Declaration of Helsinki, and the protocol was approved by the Bioethical Commission of the Pomeranian Medical University in Szczecin (permission number KB-0012/12/12).

### Assessments

The first part of this study was survey-based and conducted using the following standardized research tools: the State-Trait Anxiety Inventory (STAI) and the UWIST Mood Adjective Checklist (UMACL).

STAI consists of two parts, each including a set of 20 questions. The first part, STAI (X-1), is used to determine the level of anxiety understood as a transitory and situation-dependent status of a person. The second part, STAI (X-2), serves for measuring anxiety regarded as a rather stable personality trait (Sosnowski et al. 2006). Respondents choose one of four possible answers to each statement. The level of anxiety is expressed as the number of points obtained through summing up scores for separate answers. For each part of the questionnaire, the respondents may obtain a score of 20 to 80 points. Raw data are converted into standardized results for gender and age (stens). In our study, we used the 10-unit sten scale interpreted as follows:

- √Scores of 1–4—low results (a low level of anxiety as a trait and a state)
- √Scores of 5–6—average results (an average level of anxiety as a trait and a state)
- √Scores of 7–10—high results (a high level of anxiety as a trait and a state)

A high score for state anxiety suggests that a person is under stress caused by a difficult life situation. A high score for anxiety as a trait, on the other hand, implies a permanent predisposition to react with anxiety to life situations (Sosnowski et al. 2006).

UMACL has been developed to measure mood understood as an affective experience of moderate duration not related with the object or related with a quasi-object (Goryńska 2005). It consists of a sheet with 29 printed adjectives. UMACL is divided into three subscales:

- √Hedonic tone—10 items
- √Tense arousal—9 items
- √Energetic arousal—10 items

The raw result of each UMACL subscale is the sum of points for the items included in this subscale. The answers are weighed on a four-point scale. A raw result of the hedonic tone subscale may range from 10 to 40 points, the tense arousal subscale from 9 to 36 points, and the energetic arousal subscale from 10 to 40 points. Raw points were converted into stens by using the formula by Goryńska (2005).

The second stage of the study was based on genetic analysis, in which DNA was isolated from whole blood by a salting-out method according to Miller et al. (1988). DNA polymorphisms were identified using a polymerase chain reaction (PCR). The purpose of the analysis was to amplify the fragment which consisted of two to five repetitions of the 30-bp VNTR polymorphism in the *MAO-A* promoter region. The following primers were used F: 5′ CCC AGG CTG CTC CAG AAA 3′ and R: 5′ GGA CCT GGG CAG TTG TGC 3′. The PCR consisted of an initial denaturing step at 95 °C for 3 min, followed by 34 cycles of denaturing at 94 °C for 40 s, annealing at 57 °C for 35 s, and polymerization at 72 °C for 50 s, with a final elongation step at 72 °C for 10 min.

The sizes of the amplified fragments were as follows: 239, 209, 226, and 269 bp. In the analysis of the 5-HTT polymorphism, the fragment including the 44-bp ins/del in the regulatory sequence was amplified. The following primer sequences were used: *HTT F*, 5′ GGC GTT GCC GCT CTG AAT GC 3′; and *HTT R*, 5′ GAG GGA CTG AGC TGG ACA ACC AC 3′. The PCR consisted of an initial denaturing step at 94 °C for 5 min, followed by 30 cycles of denaturing at 94 °C for 55 s, annealing at 55 °C for 50 s, and polymerization at 72 °C for 60 s, with a final elongation step at 72 °C for 10 min. The sizes of the amplified fragments were 484 and 528 bp. The PCR products were electrophoresed on 3 % agarose gel, which was followed by ethidium bromide staining to detect the alleles (Miller et al. 1988).

FSH and AMH levels were determined as the third stage of the study. Venous blood samples were collected from women in the follicular phase of the menstrual cycle using a closed system (Vacutainer) after the women gave their consent to this procedure. The blood was drawn in the treatment room and delivered to the laboratory in accordance with the relevant rules and procedures. The levels of FSH and AMH were determined in a laboratory accredited with ISO 9001:2008 quality certification.

The FSH ranges accepted in the study as normal were FSH follicular levels, namely 3.5–12.5 mlU/ml.

### Statistical analyses

A statistical analysis was performed using STATISTICA 7.1 PL. The chi-squared independence test was employed to verify the null hypothesis about the independence of the variables. Spearman’s rank R correlation coefficient was applied to test the strength of a relationship between the ordinal variables.

The Hardy-Weinberg principle was *p* = 0.952 for *MAO-A* and *p* = 1 for 5-HTT.

The Shapiro-Wilk test for normality demonstrated the lack of a normal distribution of the variables analyzed.

Independent variables were analyzed using the Mann-Whitney *U* test.

The results were regarded as statistically significant when *p* ≤ 0.05. The level of statistical significance was set at *p* ≤ 0.05.

The level of statistical significance was set at *α* = 0.05. The power calculated for all genetic tests exceeded 0.95 (power > 0.95).

## Results

The women included in the study were aged 42.3 ± 4.5 years on average. More than half of them (75.1 %) had completed higher education; 22.6 %, secondary education; 2.0 %, vocational education; and 0.3 %, primary education. The majority of the women lived in cities with a population greater than 100,000 residents (72.5 %); 12.0 and 2.9 % lived in rural areas and towns with a population of up to 10,000, respectively; and the remainder (12.8 %) lived in towns with less than 100,000 residents. The vast majority of the participants had life partners (75.1 %).

In the analysis of anxiety as a state (STAI X1), the median was 4 stens and the interquartile range (IQR) 2 stens. In the analysis of anxiety as a trait (STAI X2), the median was the same, and the IQR was 3 stens.

In the mood analysis performed using a standardized instrument, UMACL, the median and the IQR for hedonic tone were 2 stens and 1 sten, respectively, and they were identical as for energetic arousal. The median for tense arousal was 8 stens and the IQR 1 sten.

In the study group of women, average hormone levels were as follows: E2 80 pg/ml, IQR 96.1, FSH 6.4 ml U/ml, IQR 3.5, AMH 1.33 ng/ml, IQR 2.34.

There was no statistically significant relationship between the mood (hedonic tone level, tense arousal, energetic arousal) of postmenopausal women according to UMACL and the distribution of genotypes and the frequency of alleles of the 44-bp VNTR polymorphism in the 5-HTT (*SLC 6A4*) promoter region and the 30-bp VNTR polymorphism in the *MAO-A* promoter region (*p* > 0.05) (Table 1).

**Table 1:** Analysis of the relationship between the women’s mood according to UMACL and the distribution of genotypes of the 44-bp VNTR polymorphism in the 5-HTT (*SLC 6A4*) promoter region and the 30-bp VNTR polymorphism in the *MAO-A* promoter region

UMACL (*N* = 345)	5-HTT								
	Genotypes			Depending on genotypes					
	l/l (*n* = 132)	l/s (*n* = 163)	s/s (*n* = 50)	l/l vs. l/s + s/s		s/s vs. l/l + l/s		l/l vs. s/s	
	M (IQR)	M (IQR)	M (IQR)	Z	*p*	Z	*p*	Z	*p*
TH	2 (2)	2 (2)	2 (1)	−0.445	0.656	−1.116	0.265	−1.083	0.279
PN	8 (1)	8 (1)	8 (1)	0.910	0.363	1.100	0.271	1.283	0.200
PE	2 (1)	1 (1)	2 (1)	0.287	0.774	−1.353	0.176	−0.945	0.345
UMACL (*N* = 339)	MAO-A								
	Genotypes			Depending on genotypes					
	4/4 (*n* = 142(	4/3 (*n* = 153(	3/3 (*n* = 44(	4/4 vs. 4/3 + 3/3		3/3 vs. 4/4 + 4/3		4/4 vs. 3/3	
	M (IQR)	M (IQR)	M (IQR)	Z	*p*	Z	*p*	Z	*p*
TH	2 (1)	2 (1)	2 (1,5)	0.514	0.607	0.834	0.404	0.831	0.406
PN	8 (1)	8 (1)	9 (1)	−0.207	0.836	−1.424	0.154	−1.221	0.222
PE	2 (1)	2 (1)	2 (1)	−0.726	0.468	0.072	0.942	−0.207	0.836

*N* number of participants, *n* number of participants in genotypic subgroup, *M* median, *IQR* interquartile range, *Z* Mann-Whitney *U* test statistics, *p* level of significance, *TH* hedonic tone, *PN* tense arousal, *PE* energetic arousal

Analysis of the data did not demonstrate statistically significant differences in the levels of state anxiety (STAI X1) and trait anxiety (STAI X2) according to STAI depending on the distribution of genotypes of the 44-bp VNTR polymorphism in the 5-HTT (*SLC 6A4*) promoter region and the 30-bp VNTR polymorphism in the *MAO-A* promoter region (*p* > 0.05). The 3/5 genotypes were not shown in the table (Table 2).

**Table 2:** Analysis of the relationship between anxiety as a state (STAI X1) and anxiety as a trait (STAI X2) according to STAI and the distribution of genotypes of the 44-bp VNTR polymorphism in the 5-HTT (*SLC 6A4*) promoter region and the 30-bp VNTR polymorphism in the *MAO-A* promoter region

STAI (*N* = 345)	5-HTT								
	Genotypes			Depending on genotypes					
	l/l (*n* = 132)	l/s (*n* = 163)	s/s (*n* = 50)	l/l vs. l/s + s/s		s/s vs. l/l + l/s		l/l vs. s/s	
	M (IQR)	M (IQR)	M (IQR)	Z	p	Z	p	Z	p
STAI X1	4 (2)	4 (2)	4 (2)	−0.180	0.857	−0.991	0.322	−0.877	0.381
STAI X2	4 (3)	4 (3)	4 (3)	−0.412	0.680	−0.684	0.494	−0.717	0.473
STAI (*N* = 339)	MAO-A								
	Genotypes			Depending on genotypes					
	4/4 (*n* = 142)	4/3 (*n* = 153)	3/3 (*n* = 44)	4/4 vs. 4/3 + 3/3		3/3 vs. 4/4 + 4/3		4/4 vs. 3/3	
	M (IQR)	M (IQR)	M (IQR)	Z	*p*	Z	*p*	Z	*p*
STAI X1	4 (2)	4 (2)	4 (3)	0.294	0.769	0.838	0.402	0.800	0.424
STAI X2	4 (3)	4 (3)	4 (3)	−0.007	0.994	−0.107	0.915	−0.108	0.914

*N* number of participants, *n* number of participants in genotypic subgroup, *M* median, *IQR* interquartile range, *Z* Mann-Whitney *U* test statistics, *p* level of significance

No statistically significant relationships between age and hormonal factors and mood according to UMACL and anxiety according to STAI were demonstrated (Table 3).

**Table 3:** Spearman’s rank correlation coefficient

*N* = 345	Age	AMH	FSH	E2
	R	R	R	R
Mood according to UMACL				
TH	−0.020	0.041	0.002	−0.089
PN	0.066	−0.060	0.006	0.042
PE	−0.011	0.097	−0.024	−0.035
Anxiety according to STAI				
STAI X1	0.030	−0.000	0.45	−0.095
STAI X2	−0.012	0.005	0.040	−0.042

There is no statistically significant correlation relationship between the variables analyzed in the study

*TH* hedonic tone, *PN* tense arousal, *PE* energetic arousal

## Discussion

The most frequently analysed molecules engaged in the regulation of the level of serotonin in the brain are serotonin transporter (5-HTT), responsible for the transport of this hormone from the extracellular space, and monoamine oxidase A (MAO-A), a key enzyme taking part in the degradation of serotonin. Genetic variants in genes encoding these proteins vary in transcriptional activity. Numerous scientific reports suggest that there are relationships between genetic variants in these genes, specific behavioral traits, and psychiatric disorders (Nordquist and Oreland 2010).

The *5-HTTLPR* polymorphism is the most widely investigated genetic variant used in psychiatric research (Mushtaq et al. 2014).

Sen et al. (2004) noticed that a large number of studies are based on the assumption that personality traits, such as neuroticism measured with the Neuroticism-Extraversion-Openness (NEO) Inventory and harm avoidance assessed with the Temperament and Character Inventory (TCI), are related to genetic factors. These authors conducted a meta-analysis which included a total number of 5629 participants, and found suggestive evidence for the connection between the presence of the *5-HTTLPR s* allele and lower levels of anxiety-related personality traits (*p* = 0.087). Nevertheless, they emphasize the diversity of findings concerning these issues, which mainly results from the use of different research instruments. When the analysis was stratified by inventory type, the authors noticed a strong relationship between the *5-HTTLPR* and the level of neuroticism as gauged by the NEO Inventory, while the relationship between this gene polymorphism and harm avoidance according to the TCI/Tridimensional Personality Questionnaire (TPQ) was not statistically significant.

The meta-analysis conducted by Schinka et al. (2004) showed that the level of anxiety was not related to the presence of the *s*/*s* genotype of the *5-HTTLPR* polymorphism. The same study, however, demonstrated a small but significant relationship between this polymorphism and the level of neuroticism assessed using the five-factor model developed by Costa and McCrae (Schinka et al. 2004).

Munafò et al. (2005) reported on findings that were opposite to the results of similar previous meta-analyses. They pointed to statistically significant differences in the levels of personality traits predisposing to anxiety (such as harm avoidance measured with the TCI/TPQ) in carriers of the *s*/*s* genotype and the l/l genotype of the *5-HTTLPR*. A similar relationship was not observed in the case of neuroticism assessed with the NEO Inventory. These authors believe that the relationship between the *5-HTTLPR* and anxiety-related traits cannot be excluded, but it is probably small (Munafò et al. 2005).

It was proved that carriers of the 5-HTT *s* allele were at a higher risk of anxiety disorders, but this association was only confirmed in representatives of the Caucasian race. Quite an opposite situation was observed among Asian people, whose risk of this disorder was higher if they had the ‘l’ allele (Long et al. 2013). According to other studies, being a carrier of the ‘S’ genotype raises a risk of the development of posttraumatic stress disorder (PTSD)—a type of anxiety disorder which only occurs when a person is exposed to strong environmental stress (Kilpatricik et al. 2007).

Most analyses regarding the frequency of particular genotypes in a population show that the most common is the l/s genotype. Such observation was made in the studies of Mushtaq et al. (2014) (55 % of the control group), Bellivier et al. (2000) (52.7 %), Lee et al. (2005) (65 %), and Mann et al. (2000) (57 %). In our study, a group of people with the l/s genotype was slightly smaller and included 47.25 % of all participants.

In many scientific investigations, the *s*/*s* genotype is considerably more common among people with high levels of anxiety. As many as 73.3 % of people with diagnosed anxiety disorders from India had this genotype (Mushtaq et al. 2014). Our study did not confirm a higher frequency of the *s*/*s* genotype in people with high anxiety levels—merely 32.76 % of them were its carriers. Nonetheless, it must be underlined that none of the individuals included in our study had been diagnosed as having mental disorders or had received psychiatric treatment.

The research carried out in India demonstrated that as many as 93 % of patients with anxiety disorders had either mild or moderate depression, and 40 % of patients with diagnosed depression suffered from anxiety disorders. Considering divergent reports on the association between an increased inclination to depression and the presence of the *s*/*s* genotype and the *s* allele of the *5-HTTLPR*, which either suggest that such a connection exists (Lee et al. 2005; Mann et al. 2000; Murtaza et al. 2006) or that it does not (Mushtaq et al. 2014; Margoob and Mustaque 2011; Risch et al. 2009); these issues must be further investigated.

The MAO-A enzyme, which in humans is encoded by the *MAO-A* gene, may metabolize serotonin and thus decrease its activity (Caspi et al. 2002). On a biological level, it has been proved that high activity of the *MAO-A* gene leads to the degradation of serotonin. Dysfunction of the serotonin system, on the other hand, is a part of the pathophysiology of affective disorders, including anxiety disorders (Voltas et al. 2015). It is confirmed that a functional 30-bp ins/del polymorphism in the *MAO-A* gene may be present in 2, 3, 3.5, 4, or 5 copies. The 4-repeat allele contributes to a higher *MAO-A* activity. The study conducted in 2003 by Tadic et al. (2003) was the first to prove empirically that generalized anxiety disorders are related to the presence of the *MAO-A* gene polymorphism.

*MAO-A* is regarded as an anxiety candidate gene due to efficiency of MAO inhibitors in treatment of anxiety disorders (Libert et al. 2011).

The study of 569 high school students demonstrated a significant association between the *MAO-A* gene polymorphism and anxiety levels. The results confirmed that the 4-repeat allele of the *MAO-A* gene was related to a higher level of anxiety (Liu and Lu 2013). Longitudinal research conducted in Spain on a group of 245 students confirmed a likely relationship between a variant of the *MAO-A* gene and anxiety symptoms (Voltas et al. 2015). A cohort study conducted by Chen et al. (2013) demonstrated that the low *MAO-A* expression statistically significantly increased the level of satisfaction among women.

The analysis of state anxiety and trait anxiety among premenopausal and postmenopausal women (mean age 47.7 ± 4.4) conducted in Turkey did not demonstrate statistically significant differences; nevertheless, the level of anxiety in a group of older women was slightly higher. In both groups, the level of anxiety was high (STAI-I 50.4 ± 9.1 vs. 47.5 ± 8.3; STAI-II 47.5 ± 9.1 vs. 45.4 ± 7.5 in postmenopausal and premenopausal women, respectively) (Sagsöz et al., 2001). The results reported by Pearlstein et al. (1997) show that the severity of depressive symptoms and the level of anxiety increase several years before menopause. Our study did not reveal any significant relationships between particular genotype variants of the *MAO-A* gene and the level of anxiety as a state or as a trait in a group of premenopausal women (mean age 42.3 ± 4.5 years).

The analysis of patients with diagnosed and treated mood disorders in India revealed that the most common genotype in these people was l/s (43.3 %). The l/l genotype was found in 33.3 % (Mushtaq et al. 2014). In our study, a similar distribution of genotypes was observed in healthy individuals with depressed mood: the l/s genotype was observed in 46.7 % and the *s*/*s* genotype in 39.3 % of the participants.

To determine the relationship between sex hormones and a tendency to develop anxiety and mood disorders, Albert et al. analyzed mood and the activity of the brain as a response to psychological stress. The study involved healthy, normally menstruating women in the phases of the menstrual cycle marked by high or low levels of estradiol. Significant differences in the activity of the brain in the situation of exposure to psychological stress were observed between the two groups. Women with high levels of estradiol showed significantly less deactivation in limbic regions than their counterparts with low levels of this hormone. The results show that in normally menstruating, healthy women in the premenopausal period, high levels of estradiol attenuate the brain activation changes and negative reaction to psychological stress (Albert et al. 2015). Toffoletto et al.’s (2014) review of recent studies concerning the effects of sex hormones on neural substrates of emotional and cognitive reactions suggests that sex hormones may influence cortical and subcortical regions, which results in the modulation of emotional and cognitive processes. The study described in this article did not confirm the relationship between the levels of sex hormones (AMH, FSH, and E2) and the subjective perception of mood and anxiety experienced by the participants.

## Conclusions

1. In the study, anxiety and mood were not related to the 5-HTT and *MAO-A* gene polymorphisms in healthy, late-reproductive-stage women.
2. Mood and anxiety were not significantly related to hormonal factors in the study group of healthy, late-reproductive-stage women.

### Limitations

Considering the results obtained, it seems reasonable to carry out more detailed analysis of the influence of psychological and social factors, which may be essential for the severity of anxiety and mood disorders in healthy women. The findings in this study emphasize that the relationship between anxiety and its related mood disorders is complex and require further study. We plan to continue our research in order to observe the above-described relationships. It should be emphasized that our study involved healthy women only, and though it did not confirm the connection between genetic and hormonal factors and the severity of anxiety and mood disorders, it can be used in a comparative study of women suffering from disorders.
